# Supplementary material for: Complexome profiling of the Chlamydomonas psb28 mutant reveals TEF5 as an early PSII assembly factor
Source: Plant Cell. 2025 Mar 17;37(6):koaf055. doi: 10.1093/plcell/koaf055 (PMC12164590; doi:10.1093/plcell/koaf055)
Supplement: koaf055_Supplementary_Data [file koaf055_supplementary_data.zip › psb28_interactive.zip!/plots/intensity/qualityPlots/peptides/CYN19-3_Cre13.g588100.t1.2.html]

### Cre13.g588100.t1.2

Trivial name: CYN19-3  
SeqCoverage [median]: 20.70  
Mass: 29.13 kDa  
  
 

| Sequence | MissedCleavage | Mass [Da] | wtIntensity | muIntensity | Score |
| --- | --- | --- | --- | --- | --- |
| DAVAQMAK | 0 | 832.41 | 4032.47 | 7304.76 | 134.81 |
| DNGTGGVSIYGAR | 0 | 1265.60 | 627.54 | 1603.58 | 121.61 |
| FEDENFR | 0 | 955.40 | 4117.15 | 7548.00 | 114.02 |
| HVVFGQVVEGYEVVK | 0 | 1687.89 | 6281.53 | 5337.23 | 107.65 |
| YGDATPLGR | 0 | 948.47 | 15966.37 | 13811.94 | 139.88 |
|  |  |  | 31025.1 | 35605.5 |  |
